# Specific IgE to Ara h 2 is the best predictor for peanut allergy in adults and is correlated to clinical severity

**DOI:** 10.1186/2045-7022-3-S3-P163

**Published:** 2013-07-25

**Authors:** R Klemans, H Broekman, E Knol, C Bruijnzeel-Koomen, H Otten, S Pasmans, A Knulst

**Affiliations:** 1 Dermatology/Allergology, University Medical Center Utrecht, Utrecht, the Netherlands; 2 Immunology, University Medical Center Utrecht, Utrecht, the Netherlands

## Background

Specific IgE to Ara h 2 as clinical predictor for peanut allergy in children has a diagnostic value comparable to a recently published prediction model containing gender, skin prick test (SPT), specific IgE (sIgE) to peanut extract and total IgE. The need for food challenges could be reduced by implicating both methods. In adults, the diagnostic value of peanut components has not been studied yet.

Our aim was to validate in an adult population the published prediction model and in case of poor validation, build a new model specific for adults. In addition, to define the diagnostic value of sIgE to peanut components and their correlation to clinical severity.

## Methods

Validation was performed by discrimination with an area under the receiver operating characteristic (ROC) curve (AUC) and calibration with the Hosmer-Lemeshow test and a calibration plot. The diagnostic value of sIgE to Ara h 1, 2, 3, 8 and 9 was analyzed with an ROC-curve. Sensitivity, specificity and positive and negative predictive values of both the prediction model and peanut components were calculated for different cut-off values. Correlation coefficients were calculated to analyze possible correlations between IgE to all peanut components and clinical severity.

## Results

Validation of the previously published model in 94 adult patients showed poor discrimination (AUC 0.64), but good calibration (*P* = 0 .73). Building a new model with age, SPT, specific IgE to peanut extract and Ara h 2 as candidate predictors, left only SPT in the model with an AUC of 0.72, which was lower than the AUC for sIgE to Ara h 2 as single predictor (0.76). A 95% PPV for sIgE to Ara h 2 was found at a cut-off value of (≥1.4 kU/L). By using a cut-off value with a 100% positive predictive value (≥1.8 kU/L) for sIgE to Ara h 2, 28% of patients could be accurately diagnosed. A 95% or 100% negative predictive value could not be calculated for any test. sIgE to Ara h 2 was significantly correlated to clinical severity.

## Conclusion

Discrimination of the previously published model was poor, however, calibration was good. The discriminative ability of sIgE to Ara h 2 was better than a new model, containing only SPT as predictor. sIgE to Ara h 2 could accurately diagnose peanut allergy in 28% of patients, thereby reducing the need for food challenges, but could not be used to accurately exclude a peanut allergy.

## Disclosure of interest

None declared.

